# Intra-Articular Injection of Adipose-Derived-MSC Exosomes and Hyaluronic Acid in Sheep Knee Osteoarthritic Models Enhances Hyaline Cartilage Regeneration

**DOI:** 10.3390/biomedicines13123070

**Published:** 2025-12-12

**Authors:** Auliya Akbar, Ismail Hadisoebroto Dilogo, Radiana Dhewayani Antarianto, Iqra Kousar, Angela Jennifer Tantry, Anissa Feby Canintika

**Affiliations:** 1 Department of Orthopaedics and Traumatology, Cibinong General Hospital, Faculty of Medicine, IPB University, Bogor 16914, Indonesia; 2 Department of Orthopaedics and Traumatology, Cipto Mangunkusumo Hospital, Faculty of Medicine, University of Indonesia, Jakarta 10430, Indonesia; ismailortho@gmail.com (I.H.D.); anissafeby29@gmail.com (A.F.C.); 3 Stem Cell Medical Technology Integrated Service Unit, Cipto Mangunkusumo General Hospital/Faculty of Medicine, Universitas Indonesia, Jakarta 10430, Indonesia; 4 Stem Cell and Tissue Engineering Research Center, Indonesia Medical Education and Research Institute (IMERI), Faculty of Medicine, Universitas Indonesia, Jakarta 10430, Indonesia; radiana.dhewayani@ui.ac.id (R.D.A.); iqrakousar806@gmail.com (I.K.); angela.jennifer@ui.ac.id (A.J.T.); 5 Department of Histology, Faculty of Medicine, Universitas Indonesia, Jakarta 10430, Indonesia; 6 Master’s Program in Biomedical Science, Faculty of Medicine, Universitas Indonesia, Jakarta 10430, Indonesia

**Keywords:** adipose-derived mesenchymal stem cells, cartilage, exosome, hyaluronic acid, sheep model, osteoarthritis

## Abstract

**Background**: Osteoarthritis (OA) is a degenerative joint disease characterized by cartilage damage. The limited regenerative capability of articular cartilage poses a therapeutic challenge. Adipose mesenchymal stem cell (MSC) exosomes have shown potential in regenerating cartilage structure in previous in vivo studies on small animals. This study aims to compare the effectiveness of intra-articular injections of adipose-derived MSC exosomes and hyaluronic acid (HA) on cartilage regeneration in a sheep osteoarthritis model. **Methods**: This in vivo study involved 18 male sheep that were induced to develop OA via meniscectomy. The sheep were randomized and divided into three groups: Group 1 (adipose MSC exosomes + HA), Group 2 (adipose MSC exosomes), and Group 3 (HA). Microscopic evaluation using histological scoring with the Pineda score, cartilage regeneration assessment through histochemical and immunohistochemical examinations, and microtopographic examination using a scanning electron microscope (SEM) were performed 6 weeks post-intervention. **Results**: Cartilage regeneration in the combination group (Group 1) exhibited a larger area of hyaline cartilage (Group 1 vs. Group 2 [40.38 ± 9.35% vs. 34.93 ± 2.32% vs. 31.08 ± 3.47%; *p* = 0.034]) and a smaller area of fibrocartilage compared to adipose MSC exosomes (Group 2) or HA alone (Group 3) (13.06 ± 2.21% vs. 18.67 ± 3.13% vs. 28.14 ± 3.67%; *p* = 0.037). Microtopographic examination also showed a more homogeneous and smoother cartilage surface in the combination group (Group 1) of adipose MSC exosomes and HA. **Conclusions**: In a sheep knee osteoarthritis model, intra-articular injection of a combination of adipose-derived MSC exosomes and HA significantly enhances cartilage regeneration compared to injections of adipose-derived MSC exosomes or HA alone.

## 1. Introduction

Osteoarthritis (OA) of the knee is a chronic debilitating disease that is of the entire joint organ that involves the progressive degeneration and changes in all joint tissues, including the articular cartilage, menisci, infrapatellar fat pad, synovial membrane, and subchondral bone. This comprehensive involvement often leads to significant pain and decreased quality of life for patients, which may hinder them from work and daily activities. While minor cartilage damage may be spontaneously repaired within the joint by chondrocytes, there is very little chance of a severely impaired joint regenerating itself when damaged. Therefore, any significant joint injury has little chance of healing spontaneously without some form of therapy [1]. The main treatments for osteoarthritis include conservative and surgical approaches. Conservative treatment may include a combination of pain relief medications, physical therapy, and lifestyle modifications, with joint replacement surgery considered for end-stage disease [2,3]. However, both methods only relieve symptoms and do not delay or reverse the course of osteoarthritis [4].

Extracellular vesicles (EVs) are naturally released, membrane-limited vesicles from cells which function to deliver signaling molecules within their cargo. EVs are divided into three categories, namely exosomes, microvesicles (MVs), and apoptotic bodies. Exosomes are the smallest EVs with a particle size of less than 150–200 nm. Exosomes’ contents include lipid rafts, short or long non-coding nucleic acids (lncRNA or miRNAs), and small peptide fragments [4].

Cartilage regeneration in OA is influenced by mechanical, biochemical, and inflammatory factors, leading to the formation of fibrocartilage with low mechanical quality. Mesenchymal stem cell (MSC) therapy has garnered interest as a regenerative approach. Adipose tissue, bone marrow, or umbilical cord-derived MSCs have all shown the ability to repair cartilage defects -albeit with varying degrees of success [5,6]. However, despite the promise of MSCs, there are shortcomings, such as the risk of teratoma formation and ethical issues [7]. MSCs also have stringent criteria for storage and transportation compared to exosomes, which have been shown to tolerate short-term storage at 4 °C [8,9,10]. Exosomes derived from MSCs function similarly to MSCs, replicating their key anti-inflammatory and anti-apoptotic effects. This functional overlap occurs because exosomes carry signaling molecules, i.e., miRNAs and lncRNA, that regulate chondrogenesis [11]. Therefore, exosomes, which are part of MSC secretion, have been proposed as a promising alternative due to their ability to regenerate tissues without the risk of teratoma formation and ethical issues [7,12].

While previous studies have shown promise using small animal models, there remains a critical gap in preclinical study using large animal models, which resemble the human knee joint histophysiology and the specific clinical translational targets (signal transduction pathway). This study is a continuation of the previous study by Fiolin et al. [13], whose work revealed the mechanism of action by which multiple intra-articular injections of adipose tissue-derived MSC exosomes, combined with hyaluronic acid, delivered miRs that improved chondrogenesis in the knee OA sheep model. This study aims to evaluate whether chondrogenesis induced by multiple intra-articular injections of adipose tissue-derived MSC exosomes combined with hyaluronic acid forms higher hyaline cartilage or higher fibrous cartilage in the knee OA sheep model. Evaluation methods include histological examination, immunohistochemistry, and microtopography to observe the cartilage structure formed after exosome treatment. We hope that this study will provide new insights into the development of regenerative therapies to effectively address osteoarthritis.

## 2. Materials and Methods

This was a post-test only control group experimental test on male local sheep (*Ovies aries*). The research was conducted at the Bogor Agricultural Institute, Department of Histology, Faculty of Medicine, Universitas Indonesia, Jakarta, Indonesia, the Molecular Biology and Proteomic Core Facilities (MBPCF) of Indonesia Medical Education and Research Institute, Faculty of Medicine, Universitas Indonesia, Jakarta, Indonesia, and Stem Cell and Tissue Engineering (SCTE), Faculty of Medicine, Universitas Indonesia, Jakarta, Indonesia. The animal study protocol was approved by the Ethics Committee of the Faculty of Medicine, Universitas Indonesia, Jakarta, Indonesia, with ethical approval number KET-932/UN2.F1/ETIK/PPM.00.02/2022, dated 5 September 2022, with validity for one year. In addition, animal experimentation complied with ARRIVE guidelines as the protocol has been approved by the Animal Care and Use Committee (ACUC), School of Veterinary Medicine and Biomedical Sciences, IPB University, number 023/KEH/SKE/IX/2022.

### 2.1. Eligibility Criteria

The study samples were selected based on specific inclusion and exclusion criteria. The inclusion criteria required male local sheep (*Ovies aries*) aged over 3 years, weighing between 25 and 30 kg, and showing no abnormalities in the lower extremities, including congenital issues or trauma. Additionally, the sheep needed to be skeletally mature, as confirmed through radiological examination. Exclusion criteria included death during the study, infection in the knee joint under investigation, the development of lower extremity disorders during the study period, or the presence of cartilage abnormalities discovered during meniscectomy surgery. The selected samples were then randomly allocated into three treatment groups using the simple random sampling method: Administration of adipose-derived MSC exosome injection, administration of hyaluronic acid (HA) injection, and administration of a combination of adipose-derived MSC exosome injection and HA. Samples were taken randomly from male *Ovies aries*. Sample size was estimated using the resource equation: E = total number of animals − total number of groups, with E estimated between 10 and 20. This value is consistent with the ‘principle of least animals’ used in preclinical trials to ensure the ethical use of large animal models.

### 2.2. Adipose-Derived MSC Exosome Preparation

Cryoprecipitate of secretome adipose-derived MSC from human was collected and kept at −20 °C freezer GPS F700-SAEV-TS (Thermo Fisher Scientific, Singapore) in SCTE IMERI lab, Faculty of Medicine Universitas Indonesia (FMUI). The frozen conditioned medium (CM) was defrosted by submerging its container in room temperature water at SCTE IMERI FMUI. In Fiolin et al. [14], MSC-EVs were isolated through a multi-step density ultracentrifugation protocol. The first step involved centrifugation at 750× *g* and 2000× *g* for 15 min using the S-4-72 swing-bucket rotor (Eppendorf, Enfield, CT, USA) and 50 mL tubes. The second step was performed at 10,000× *g* for 45 min using a 24 × 1.5/2.0 mL rotor with ClickSealTM at a 45° angle (Thermo Scientific, Singapore), followed by filtration through a 0.22 µm filter. Finally, MSC-EVs were isolated by ultracentrifugation at 100,000× *g* for 90 min using a T-865 fixed-angle rotor at a 23.5° angle (Thermo Scientific, Singapore). After that, the pellet containing the exosome was transferred to a 15 mL Falcon tube, and the supernatant was discarded. After adding cold Dulbecco’s Phosphate-Buffered Saline (D-PBS) (14200075, Gibco, Waltham, MA, USA) till the volume reached 5 mL, the mixture was re-dissolved. The exosomes were separated into a 1 mL cryovial and stored for up to three months in either −80 °C freezer UF V 500 (Binder, Tuttlingen, Baden-Württemberg, Germany) or −20 °C freezer GPS F700-SAEV-TS (Thermo Scientific, Singapore). Isolated adipose tissue-derived MSC-Exos were characterized for size, polydispersity index (PI), and zeta potential (ZP) using a Horiba-SZ 100z particle size analyzer (HORIBA Ltd., Kyoto, Japan) at a constant temperature of 25 ± 1 °C and a scattering angle of 90°, following the manufacturer’s protocol. Data were obtained from three replicates. BD FACSAriaTM III flow cytometer (BD Biosciences, San Jose, CA, USA) was used to evaluate the purity of adipose tissue-derived MSC-Exos by detecting the expression of the specific marker CD 81-PE conjugated flow cytometry antibody after a prior enrichment step with CD63 capture beads incubation (Abcam). Sterility testing was performed using a VITRO respiratory flow chip kit (Vitro S.A., Seville, Andalusia, Spain) according to established procedural guidelines. The exosome showed a mean size of 88.7 nm, 40 nm standard deviation (SD) [14], zeta potential of −1.4 mV and −10.2 mV, and conductivity was between 14.945 mS/cm and 14.982 mS/cm, and negative for specific pathogens listed in the in vitro respiratory flow chip kit.

### 2.3. Hyaluronic Acid Solution

Hyaluronic acid solution was derived from Durolane™ (Bioventus, Durham, NC, USA), which is a high-viscosity hyaluronic acid (HA) containing 20 mg of sodium hyaluronate per mL.

### 2.4. Surgical Procedure and Intraarticular Injection Procedure

The study involved 18 *Ovies aries* sheep weighing between 25 and 30 kg, which were acclimatized in a laboratory environment for two weeks under controlled conditions of temperature (28–30 °C) and humidity (55%). Subsequently, unilateral total lateral meniscectomy was performed on the right hind knee of all sheep. The surgical procedure included premedication with Amoxicillin at a dose of 5 mg/kg body weight intramuscularly, and atropine sulfate 0.15 mg/kg body weight, followed by induction with ketamine 22 mg/kg body weight intramuscularly, and muscle relaxant xylazine 0.2 mg/kg body weight was administered. Surgery was conducted using a lateral parapatellar surgical approach, involving incision, dissection, and removal of the lateral meniscus. The surgical site was irrigated with saline and closed layer by layer [15].

Following the surgery, the sheep were housed in cages for 10 days until the surgical wounds healed. After a 10-day observation period, stitches were removed, and the sheep were trained to walk again. They underwent a three-week training period involving walking on asphalt.

Post-meniscectomy, the sheep were prepared for injections of hyaluronic acid, adipose tissue-derived MSC exosomes, or a combination of both into the right knee. Injections were administered to six sheep per intervention group, with aseptic precautions taken. Each sheep received a 2 mL injection of hyaluronic acid, exosomes, or their combination. Sheep were then monitored for one month post-injection before euthanasia was performed for further analysis.

### 2.5. Histological Exam

Cartilage samples were taken from the femoral condyle and trochlear groove. The FFPE blocks were prepared as described previously [16], involving fixation in 10% formalin, followed by electrolytic decalcification (90% formic acid, HCl, and distilled water made in Histology lab FMUI) and subsequent dehydration by increasing concentrations of EtOH (70%, 80%, 95%, absolute), clearing using xylene, and embedded in paraffin wax. For histological examination, FFPE blocks were cut into 5-µm-thick sections serially using a rotary microtome (Leica Biosystem, Nussloch, Germany), followed by hematoxylin-eosin staining. Hematoxylin-eosin staining was performed using the methodology previously outlined [15]. Briefly, slides were deparaffinized in xylene and rehydrated through a graded EtOH series (absolute, 95%, 80%, 70%) to ddH_2_O. The cell nuclei were stained by incubating in Harris’s hematoxylin (3801562, Leica, Buffalo Grove, IL, USA) for 5 min, followed by a water rinse. Eosin Y (3801602, Leica, Buffalo Grove, IL, USA) stains cytoplasmic and extracellular matrix through 30 s dips. Histology slides were dehydrated using graded EtOH concentrations (70%, 80%, 95%, absolute) and cleared twice in fresh xylene solution. The slides were mounted with entellan (107960, Sigma-Aldrich, Bavaria, Germany) and a cover slip.

Masson Trichrome staining was also performed to evaluate the percentage of fibrous areas. In brief, FFPE slides were deparaffinized in xylene and rehydrated using decreasing graded EtOH and ddH_2_O. Immediately after rehydration, the slides were secondary fixed in Bouin’s solution for 1 h at 56 °C, then left to cool at room temperature before washing with running water to remove the remaining Bouin’s solution. Counterstaining was performed by incubating the slides in Eisenhematoxylin–Weigert solution for 7 min. Followed by washing with running water for 10 min and then soaked in ddH_2_O for 3 min, and air-dried. After that, the slides were stained with Biebrich Scarlet-acid fuchsin for 10 min, rinsed with ddH_2_O for 1 min, and incubated in phosphotungstic-phosphomolybdic acid for 15 min. The FFPE slides were then incubated in aniline blue for 10 min, rinsed with ddH_2_O for 5 min, and immersed in 1% acetic acid for 5 min. The process was ended by clearing and mounting.

The Area of Hyaline cartilage, fibrocartilage, and amorphous substance was performed using safranin O fast green (SAFO) staining at the Histology Lab FMUI. SAFO staining began with deparaffinization in xylene I, xylene II, and xylene III for five minutes each. Rehydration was performed with 95% alcohol (twice) and 70% alcohol for one minute each, then rinsed in water for one minute. The specimen was placed in Weigert’s hematoxylin (hematoxylin, 95% alcohol, ferric chloride, HCl, and distilled water made in the Histology Lab FMUI) for 15 min and rinsed with running water for 5 min. The fast green FCF solution is prepared in the Histology Lab FMUI by mixing 0.04 g of fast green FCF with 100 mL of distilled water. The acetic acid solution is prepared in the Histology lab FMUI by mixing 1 mL of glacial acetic acid with 99 mL of distilled water. The safranin O solution is prepared in the Histology Lab FMUI by mixing 0.1 g of safranin O with 100 mL of distilled water. The specimen was placed in a Fast Green FCF solution for 25 min, dipped twice in acetic acid solution, and dipped in safranin O solution for 15 min. After that, dehydration was performed again in 95% alcohol (twice) and 100% alcohol, each with several dips. The specimen is placed in xylene for five minutes, then covered with entellan and sealed with a cover slip.

For each slide, fields of view were captured at 10× (numerical aperture 0.25, working distance 4.39 mm) and 40× (numerical aperture 0.65, working distance 0.48 mm) objective lens magnifications using a ZEISS Primostar 1 light microscope (Carl Zeiss AG, Oberkochen, Baden-Württemberg, Germany) equipped with a Beta Sony Exmor CMOS Sensor Camera (Indomicro, Jakarta Timur, Indonesia; 3.1-megapixel resolution), connected to a computer at Dry Lab E410 RIK UI Depok. The entire field of view was microphotographed systematically using a top-down and sequential approach. Scale bars were added automatically in the micro-photographs using the IndomicroView camera-compatible computer program version 3.7 (Indomicro, Jakarta Timur, Indonesia). Microphotographs with a resolution of 1920 × 1080 pixels (24-bit RGB 3 channels) were stored as PNG files. Several microphotographs were stitched together using ImageJ with the Fiji version plugin to display the whole cartilage lesion or regeneration. Cartilage repair was scored using the Pineda scoring system to assess filling defect, osteochondral junction, matrix staining, and cell morphology, and area percentage was calculated using ImageJ https://imagej.net/software/fiji/ (accessed on 27 April 2024).

### 2.6. Immunohistochemistry Exam

Immunohistochemistry for type III collagen was performed to examine the extracellular matrix components. Immunohistochemistry for type III collagen used a rabbit polyclonal anti-type III collagen primary antibody (Abcam antibody, ab7778, concentration: 1:750). Immunohistochemistry for biglycan using a mouse monoclonal anti-biglycan primary antibody (Santa Cruz antibody, sc-100857, concentration: 1:1000). The secondary antibody used was mouse m-IgGk BP-HRP secondary antibody (Santa Cruz antibody, sc-516102, concentration: 1:25).

Immunohistochemistry for type III collagen and byglycan was performed by deparaffinizing the preparations with xylol for 2 × 15 min. The preparations were rehydrated with graded alcohol from 100%, 95%, 80%, and 70% alcohol for 5 min each. The preparations were then placed in distilled water for 5 min. Wash the preparation with PBS 1× for 2 × 5 min. Add a hydrogen peroxide block to the preparation for 10 min. Wash the preparation with PBS 1× for 3 × 5 min. Block the preparation with a protein block for 30 min. Wash the preparation with PBS 1× for 1 × 5 min. The specimen is incubated with the primary antibody in a moist chamber in a refrigerator at 4 °C overnight. Then, the specimen is washed with PBS 1× for 3 × 5 min. The specimen is incubated with HRP-conjugated rabbit secondary antibody for 1 h. The specimen is washed with PBS 1× for 3 × 5 min. The specimen is stained with 3-3’-diaminobenzidine (DAB) for 1 min. The specimen is washed with running water. DAB preparation is performed with a DAB chromogen to DAB substrate ratio of 1:50. The specimen is counterstained with Harris hematoxylin for 30 s. The specimen is washed with running water. The specimen is treated with lithium carbonate for 30 s. The specimen is washed with running water. The specimen is dehydrated with graded alcohol solutions of 70%, 80%, 95%, and 100% alcohol, each for 5 min. Clearing of the specimen is performed by immersing it in xylene for 2 × 5 min. A cover slip is placed on the slide, and the cover glass is sealed to the slide. All these protocols were performed in the Histology Lab FMUI.

The steps in histomorphometry data analysis were performed using ImageJ and are given in Supplementary Figures S1–S3.

### 2.7. Microtopography Evaluation

We use a digital scanning electron microscope (SEM, Prisma E.M., Thermo Fisher Scientific, Waltham, MA, USA) to evaluate the ultrastructure of the cartilage surface formed in the samples. We observed the cartilage surface in 65×, 350×, and 5000× magnification. SEM examination was performed in the Integrated Laboratory of Bioproduct (iLab = N) by Badan Riset dan Inovasi Nasional (BRIN).

### 2.8. Statistical Analysis

We analyzed the accumulated data using IBM SPSS Statistics version 26.0 (IBM Corp., Chicago, IL, USA). Data was analyzed descriptively and underwent normality tests. The central and trend measures of the distribution are presented for characteristics with a normal distribution (Mean ± SD) and those with a non-normal distribution (Median; Q1–Q3). Hypothesis test was performed by comparing the mean score of the Pineda scale in the histological exam with the immunohistochemistry exam result using one-way ANOVA or Kruskal–Wallis test. Post hoc test and homogeneity test were also executed.

## 3. Results

A total of 18 sheep were included in the study, with a mean pre-meniscectomy weight of 29.58 ± 1.26 kg and a post-injection weight of 32.46 ± 0.42 kg. Most sheep had OA grade I (69.2%) as shown in Table 1.

The highest median Pineda score was in the HA group (13.5, min-max: 4–14), and the lowest was in the exosome-only group (5, min-max: 5–9). There were no significant differences between the three groups in Pineda scores post-treatment (*p* > 0.05), as shown in Table 2.

Quantitative analysis of collagen area using Masson trichrome staining and ImageJ showed no significant difference between groups (*p* = 0.412). SAFO staining revealed the highest percentage of hyaline cartilage area in the combination group (40.38 ± 9.35%, *p* = 0.034) and the lowest fibrocartilage area (13.06 ± 2.21%, *p* = 0.037). There was no significant difference in amorphous substance area between groups (*p* = 0.251). These results were summarized in Table 3.

**Figure 1:**
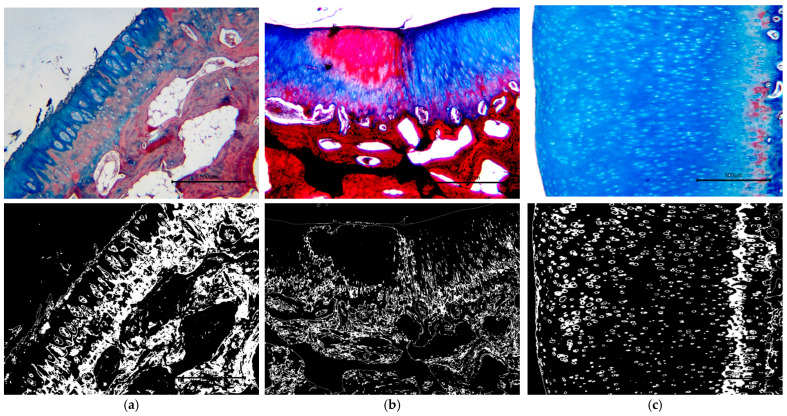
Masson Trichrome staining of sheep cartilage specimens treated with (**a**) HA; (**b**) exosome; and (**c**) a combination of both. Areas with the most distinct homogenous blue colors predominantly display hyaline cartilage, as shown in the combination group (**c**). In contrast, areas with patches of blue colors interspersed with pale red (**a**) and bright red colors interspersed with higher intensity of blue colors between the chondrocyte clusters (**b**) showed the presence of fibrous cartilage areas or lesser areas or hyaline cartilage.

**Figure 2:**
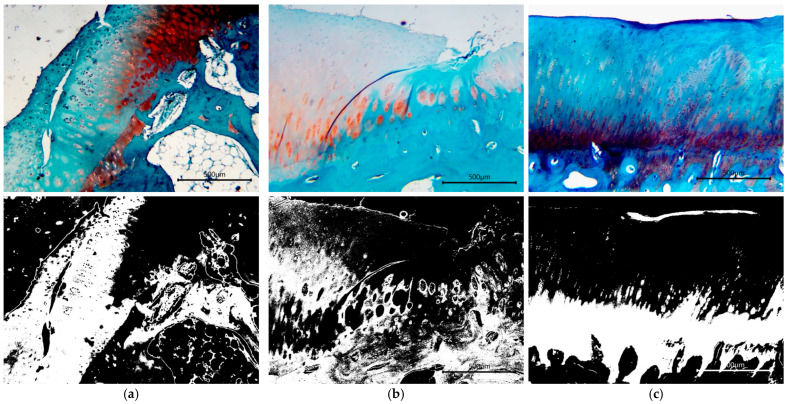
SAFO staining of sheep cartilage specimens treated with (**a**) HA; (**b**) exosome; and (**c**) a combination of both. Areas with the most distinct turquoise colors predominantly display hyaline cartilage, as shown predominantly in the combination group (**c**), while areas with patches of bright orange stained (**a**) and light orange stained areas interspersed with lower intensity of torques between the chondrocyte clusters (**b**) showed the presence of fibrous cartilage areas or lesser areas of hyaline cartilage.

Figure 1 and Figure 2 illustrate the comparative results of Masson’s Trichrome and SAFO staining, respectively, conducted on groups with HA injection, exosome injection, and combined injection. The images in the right column were the converted microphotographs of histological staining with Masson’s Trichrome after being separated based on the red color filter (to indicate the extracellular matrix composition in the form of collagen fibers) and threshold adjustments based on the original microphotographs.

**Figure 3:**
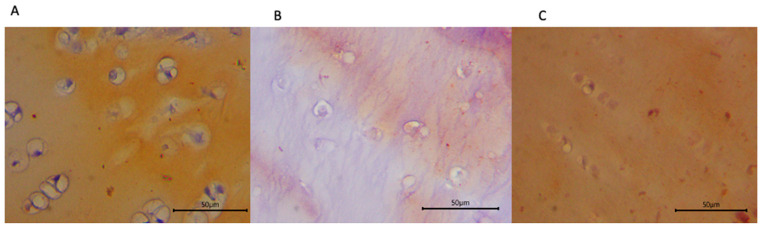
Immunohistochemical staining of sheep cartilage specimens for biglycan treated with (**A**) HA; (**B**) exosome; and (**C**) a combination of both. Note that the fraction of biglycan appears lower in the group with exosome injection alone.

**Figure 4:**
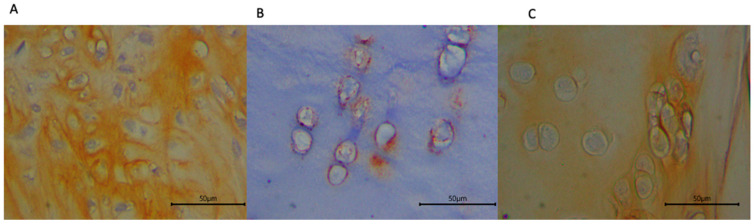
Immunohistochemical staining of sheep cartilage specimens for type III collagen treated with (**A**) HA; (**B**) exosome; and (**C**) a combination of both. Note that the fraction of type III collagen appears lower in the group with exosome injection alone.

Quantification of collagen type III and biglycan expression (Table 4) showed higher biglycan area fraction in the HA group, followed by the combination group and the exosome group (*p* = 0.372) (Figure 3). Collagen type III area fraction was higher in the combination group, followed by the HA and exosome groups (*p* = 0.478) (Figure 4).

**Figure 5:**
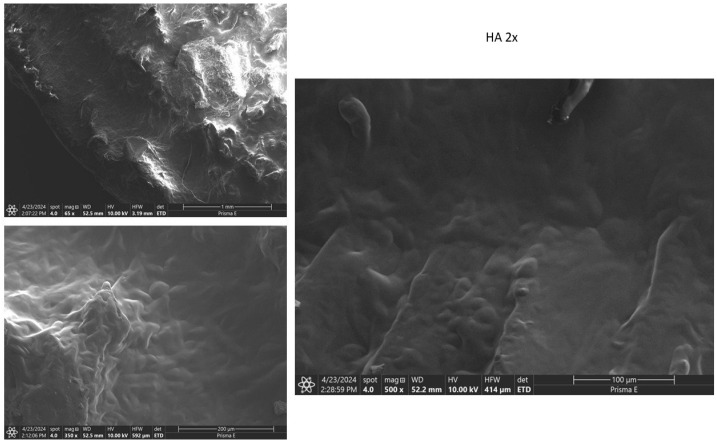
SEM examination result for HA at 65× Magnification, 350× Magnification, and 500× Magnification. Note the cartilage defect shown in the cartilage surface by an uneven and heterogeneous microtopography.

**Figure 6:**
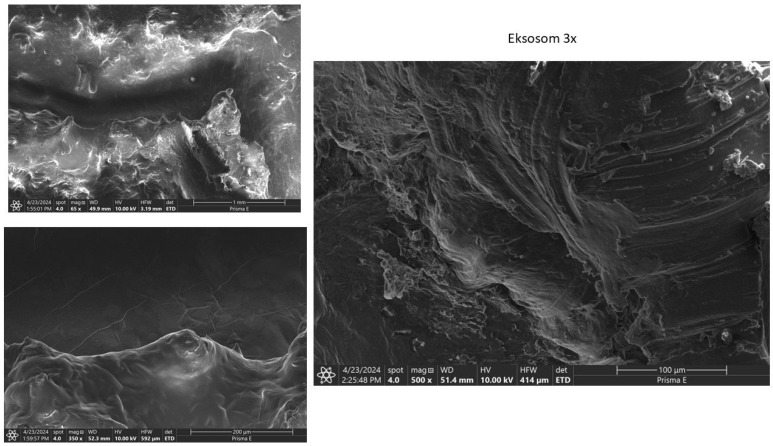
SEM examination result for exosome at 65× Magnification, 350× Magnification, and 500× Magnification. Note that the cartilage surface was more homogenous than the HA group, although still uneven.

**Figure 7:**
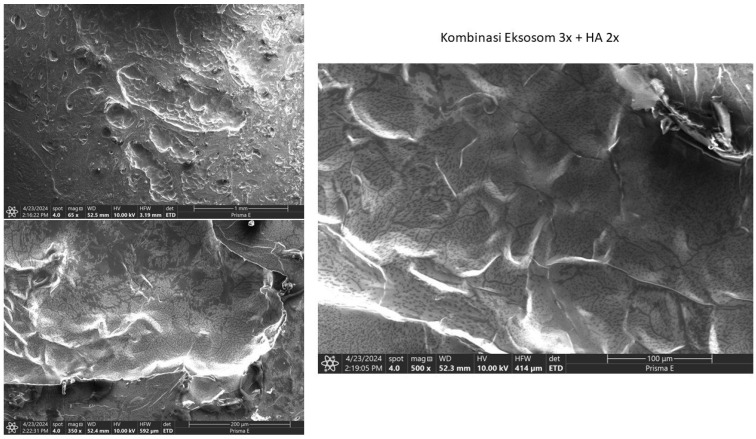
SEM examination result for the combination group at 65× Magnification, 350× Magnification, and 500× Magnification. Note that the microtomography is smooth and homogeneous.

SEM examination of femoral condyles showed partial cartilage regeneration with uneven distribution in the HA group, as shown in Figure 5. The exosome group showed partial cartilage regeneration with less homogeneous distribution, as shown in Figure 6. Meanwhile, the combination group showed extensive and evenly distributed cartilage regeneration, with a homogeneous and smooth surface (Figure 7).

## 4. Discussion

This study’s results showed that group 3, which received HA injections alone, had the highest Pineda score, with a median score of 13.5 [7,12,13,14,15,16,17,18,19,20,21,22,23,24], whereas the group that received exosome injections had the lowest Pineda score, with a median score of 5 [12,13,14,15,16,17,18,19]. There was no significant difference among the three study groups in terms of Pineda scores after treatment, with a *p*-value of 0.331.

These findings differ from previous studies. The model used in this study was a sheep model, which contrasts with the studies by Wong et al. [25], He et al. [26], and Sun et al. [27], which used small animal models, including rabbits and mice, to study the effect of exosomes in cartilage regeneration and OA progression. This difference in models could account for the varying results. In OA animal model studies, using large animal models like sheep is preferable for evaluating therapeutic effects because the anatomy, histology, and physiology of humans are more closely aligned with those of larger animals. Consequently, this study is more effective than previous studies in interpreting the therapeutic effects of exosomes [28].

In this study, the adipose MSC exosome and HA injection group had the highest percentage of hyaline cartilage area, followed by the adipose MSC exosome group, and finally the HA group (*p* = 0.034). Conversely, the highest fibrocartilage area was observed in the HA injection group, followed by the adipose MSC exosome group, with the lowest fibrocartilage area in the combination group of adipose MSC exosome and HA injections. These findings indicate that the combination of adipose MSC and HA significantly enhances cartilage regeneration. These results are consistent with previous studies showing that exosomes or MSCs play a significant role in inducing cartilage regeneration in OA animal models. This study confirms the findings from earlier research conducted on small animal models of OA [29].

In this study, the best results were obtained when adipose-derived exosomes were combined with HA. HA hydrogels are absorbable biological scaffolds composed of three-dimensional hydrophilic polymers. HA hydrogels provide excellent biocompatibility and create a suitable nutritional environment for the growth of endogenous cells. Therefore, the combination of exosomes and HA hydrogels can increase the stability and maintain the biological activity of exosomes. This study found that combining adipose-derived exosomes with hyaluronic acid (HA) yielded the best results. HA hydrogels, composed of three-dimensional hydrophilic polymers, serve as absorbable biological scaffolds that offer excellent biocompatibility and create an optimal environment for the growth of endogenous cells. Consequently, the combination of exosomes and HA hydrogels enhances exosome stability and preserves their biological activity. Additionally, HA injections have been associated with increased synovial fluid viscosity, which can act as a lubricant within the joint cavity, reducing cartilage wear and protecting joint cartilage.

In this study, the combination therapy and HA groups exhibited a larger area fraction of biglycan or amorphous substance and type III collagen compared to the exosome-only group. This suggests that the combination therapy effectively stimulates cartilage regeneration, even though the specific type of regeneration could not be specified. Moreover, HA has been widely studied for its potential to promote cartilage regeneration through its use as a scaffold. HA hydrogels have been identified as promising materials for cartilage tissue engineering due to their ability to stimulate endogenous HA synthesis, reduce joint friction, and lower the risk of damage. These hydrogels, when used as scaffolds, have been shown to synergistically modulate scaffold properties, demonstrating their potential for cartilage regeneration. Furthermore, HA-based gels have been recognized as useful drug carriers for controlling the release of growth factors, which are crucial for cartilage regeneration [30,31]. The use of HA in scaffolds has also been linked to better in situ inductive regeneration of cartilage defects, making it a desirable material for cartilage regeneration [32,33,34].

In this study, cartilage regeneration was extensive and evenly distributed. The tissue microtopography was much more homogeneous, with a smooth surface. The tissue structure distribution was relatively even, with minimal differences in surface height. Differentiation between connective tissue and cells was easily identifiable. The findings in the combination group showed better results compared to the HA and exosome groups [35,36,37].

The microtopographic changes in cartilage associated with OA involve complex interactions between cartilage and subchondral bone. Although OA was traditionally considered a cartilage disorder, it is now recognized that subchondral bone remodeling plays a significant role in disease progression. Studies have shown that microstructural changes in subchondral bone precede cartilage damage and are crucial for OA development. Furthermore, significant correlations between changes in cartilage and subchondral bone material properties have been observed during aging and OA progression, supporting the role of bone in disease initiation and progression. Abnormal mechanical pressure triggers disrupted metabolism in osteoblasts, leading to increased expression of degradative metalloproteinases and matrix metalloproteinases, contributing to microstructural and histopathological changes in subchondral bone associated with OA. Additionally, bone and cartilage interactions are heightened in OA, exacerbating bone and cartilage changes. This is further supported by findings that osteoporosis increases cartilage damage in combined osteoporosis and OA models [38,39,40,41,42].

Treating OA with exosomes and stem cells plays a role in addressing ultrastructural topographic changes in cartilage associated with the condition. Cosenza et al. [43] conducted a study on an OA mouse model induced with collagenase, which was injected with bone marrow MSCs, bone marrow MSC exosomes, and bone marrow MSC microparticles. This study examined the treatment effects on the OA cartilage surface. Electron microscopy examination showed that bone marrow MSCs, bone marrow MSC exosomes, and bone marrow MSC microparticles improved OA cartilage regeneration compared to untreated controls. Jin et al. [44], in an OA mouse model injected with bone marrow MSC exosomes, also reported similar results in OA cartilage surface examination using micro-CT. Although this study used a different exosome source than previous studies, it showed similar results, demonstrating the potential of adipose-derived MSC exosomes in improving the ultrastructure of OA cartilage. This improvement occurred through multiple pathways, such as enhanced chondrocyte markers, inhibition of inflammatory markers, and protection against apoptosis [43,45].

The strengths of this study include being the first to evaluate the effects of exosomes in a large animal model, specifically sheep. The results demonstrate good regenerative outcomes from the combination of exosomes and HA in an OA animal model. However, the limitations of this study include the lack of blinding during the evaluation of histological results. The Pineda score is a semi-quantitative assessment used to evaluate the validated histological features of cartilage. Therefore, even without blinding, the outcome assessment can be fairly objective. Additionally, the microtopographic examination in this study is subjective, which may lead to biased interpretations. The study was also conducted using only one dosage, which prevents the exploration of dose effects on cartilage regeneration outcomes. Confounders are minimized by keeping all subjects and samples in the same environment.

## 5. Conclusions

The combination of adipose-derived MSC exosomes + HA could offer hyaline cartilage regeneration; in fact, the hyaline cartilage regeneration is superior compared to exosomes or HA alone in a sheep OA model. Further studies should analyze additional parameters, including IHC for collagen types I and II, and conduct long-term follow-up studies to determine the optimal therapy duration and dose frequency for cartilage regeneration in OA.

## Figures and Tables

**Table 1:** Demographic data of the subjects based on weight and OA type.

Variables	Group 3 (HA Injection, *n* = 6)	Group 2 (Exosome Injection, *n* = 6)	Group 1 (Exosome Injection + HA, *n* = 6)	Mean Weight ± SD
Weight (kilogram)				
Pre-meniscectomy	29.32 ± 1.14	32.21 ± 1.12	28.44 ± 1.56	28.44 ± 1.56
2 weeks post-meniscectomy	31.11 ± 0.78	32.43 ± 0.98	30.97 ± 0.76	31.35 ± 0.86
4 weeks post-meniscectomy	32.12 ± 0.22	34.23 ± 0.53	32.15 ± 0.50	32.46 ± 0.42
Grade of osteoarthritis Grade I Grade II	4 (66.7%) 2 (33.3%)	3 (50%) 3 (50%)	4 (66.7%) 2 (33.3%)	

SD = standard deviation.

**Table 2:** Pineda Score comparison between groups.

Group	Pineda Score	
	Median (Min–Max)	*p*-Value
Group 1 (Exosome + HA Injection)	6 (4–10)	0.331
Group 2 (Exosome Injection)	5 (5–9)	
Group 3 (HA Injection)	13.5 (4–14)	

**Table 3:** Comparison between groups in the immunohistochemistry exam.

Groups	Area Percentage			
	Collagen Regeneration Area	Hyaline Area	Fibrocartilage Area	Amorf Substance Area
Group 1 (Exosome + HA Injection)	17.86 ± 2.41	40.38 ± 9.35	13.06 ± 2.21	29.373 ± 5.05
Group 2 (Exosome Injection)	20.84 ± 1.65	34.93 ± 2.32	18.67 ± 3.13	32.31 ± 2.35
Group 3 (HA Injection)	19.44 ± 5.29	31.08 ± 3.47	30.23 ± 2.52	28.14 ± 3.67
*p*-value between groups	*p* = 0.412	*p* = 0.034	*p* = 0.037	*p* = 0.251

**Table 4:** Comparison between groups in the immunohistochemistry exam regarding biglycan and collagen III fraction.

Group	% Biglycan Fraction Area		% Collagen III Fraction Area	
	Median (Min–Max)	*p*-Value	Median (Min–Max)	*p*-Value
Group 1 (Exosome + HA Injection)	98.11 (90.66–100)	0.372	98.63 (75.33–99.92)	0.78
Group 2 (Exosome Injection)	49.81 (25.62–73.99)		81.21 (48.46–97.72)	
Group 3 (HA Injection)	99.93 (99.83–100)		94.42 (92.34–99.1)	

## Data Availability

Raw data will be provided by request to the corresponding author in the form of an Excel spreadsheet.

## Supplementary Materials

The following supporting information can be downloaded at: https://www.mdpi.com/article/10.3390/biomedicines13123070/s1, The Supplementary Figures show steps in histomorphometry data analysis. Figure S1: Characterization of Adipose SPM Exosomes Using Flow Cytometry CD63 and CD81 in unstained sample. Figure S2: Characterization of Adipose SPM Exosomes Using enrichment of CD63 antibody and CD81 antibody Flow Cytometry. Figure S3: Characterization of Adipose SPM Exosomes Based on Particle Size with PSA.

## Abbreviations

The following abbreviations are used in this manuscript:

EV	Extracellular Vesicle
HA	Hyaluronic Acid
HE	Hematoxylin and Eosin
MSC	Mesenchymal Stem Cell
MV	Microvesicle
MBPCF	Molecular Biology and Proteomic Core Facilities
OA	Osteoarthritis
SAFO	Safranin O Fast Green
SEM	Scanning Electron Microscope
SCTE	Stem Cell and Tissue Engineering

## References

[1] Thoene M., Bejer-Olenska E., Wojtkiewicz J. (2023). The Current State of Osteoarthritis Treatment Options Using Stem Cells for Regenerative Therapy: A Review. Int. J. Mol. Sci..

[2] Roseti L., Desando G., Cavallo C., Petretta M., Grigolo B. (2019). Articular Cartilage Regeneration in Osteoarthritis. Cells.

[3] Grässel S., Muschter D. (2020). Recent Advances in the Treatment of Osteoarthritis. F1000Research.

[4] Ni Z., Zhou S., Li S., Kuang L., Chen H., Luo X., Ouyang J., He M., Du X., Chen L. (2020). Exosomes: Roles and Therapeutic Potential in Osteoarthritis. Bone Res..

[5] Kern S., Eichler H., Stoeve J., Klüter H., Bieback K. (2006). Comparative Analysis of Mesenchymal Stem Cells from Bone Marrow, Umbilical Cord Blood, or Adipose Tissue. Stem Cells.

[6] Li C., Wu X., Tong J., Yang X., Zhao J., Zheng Q., Zhao G., Ma Z. (2015). Comparative Analysis of Human Mesenchymal Stem Cells from Bone Marrow and Adipose Tissue Under Xeno-Free Conditions for Cell Therapy. Stem Cell Res. Ther..

[7] Malchau H., Herberts P., Eisler T., Garellick G., Söderman P. (2002). The Swedish total hip replacement register. J. Bone Jt. Surg..

[8] Hanna J., Hubel A. (2009). Preservation of Stem Cells. Organogenesis.

[9] Zhang K., Cheng K. (2023). Stem Cell-Derived Exosome Versus Stem Cell Therapy. Nat. Rev. Bioeng..

[10] Ge Q., Zhou Y., Lu J., Bai Y., Xie X., Lu Z. (2014). MiRNA in Plasma Exosome Is Stable under Different Storage Conditions. Molecules.

[11] Arabpour M., Saghazadeh A., Rezaei N. (2021). Anti-Inflammatory and M2 Macrophage Polarization-Promoting Effect of Mesenchymal Stem Cell-Derived Exosomes. Int. Immunopharmacol..

[12] Reissis D., Tang Q.O., Cooper N.C., Carasco C.F., Gamie Z., Mantalaris A., Tsiridis E. (2016). Current Clinical Evidence for the Use of Mesenchymal Stem Cells in Articular Cartilage Repair. Expert Opin. Biol. Ther..

[13] Wu Y., Li J., Zeng Y., Pu W., Mu X., Sun K., Peng Y., Shen B. (2022). Exosomes Rewire the Cartilage Microenvironment in Osteoarthritis: From Intercellular Communication to Therapeutic Strategies. Int. J. Oral Sci..

[14] Fiolin J., Dilogo I.H., Antarianto R.D., Pontoh L.A. (2022). Isolation and characterization of adipose-derived mesenchymal stem cell exosomes: An in-vitro study. J. Profesi Med. J. Kedokt. Kesehat..

[15] Fiolin J., Priosoeryanto B.P., Isyani T., Antarianto R.D., Prasetyo M., Harahap A.R., Pakasi T.A., Dilogo I.H., Pontoh L.A. (2024). Early low-grade knee osteoarthritis in sheep (*Ovis aries*) after 6-weeks of total unilateral meniscectomy: A radiographic evaluation. Int. J. Vet. Sci..

[16] Pontoh L.A., Fiolin J., Dilogo I.H., Prasetyo M., Antarianto R.D., Harahap A., Tantry A.J., Pakasi T.A., Priosoeryanto B.P., Dewi T.I. (2025). Combined exosome of adipose-derived mesenchymal stem cell and hyaluronic acid delays early osteoarthritis progression of ovine sheep model: Clinical, radiographic, macroscopic and microscopic evaluation. F1000Research.

[17] Lee Y.-H., Park H.-K., Auh Q.-S., Nah H., Lee J.S., Moon H.-J., Heo D.N., Kim I.S., Kwon I.K. (2020). Emerging Potential of Exosomes in Regenerative Medicine for Temporomandibular Joint Osteoarthritis. Int. J. Mol. Sci..

[18] López-Ruiz E., Jiménez G., Álvarez de Cienfuegos L., Antich C., Sabata R., Marcha J., Gálvez-Martín P. (2019). Dvances of Hyaluronic Acid in Stem Cell Therapy and Tissue Engineering, Including Current Clinical Trials. Eur. Cell Mater..

[19] Feng C., Luo X., He N., Xia H., Lv X., Zhang X., Li D., Wang F., He J., Zhang L. (2018). Efficacy and Persistence of Allogeneic Adipose-Derived Mesenchymal Stem Cells Combined with Hyaluronic Acid in Osteoarthritis After Intra-Articular Injection in a Sheep Model. Tissue Eng. A.

[20] Mobasheri A., Csaki C., Clutterbuck A.L., Rahmanzadeh M., Shakibaei M. (2009). Mesenchymal Stem Cells in Connective Tissue Engineering and Regenerative Medicine: Applications in Cartilage Repair and Osteoarthritis Therapy. Histol. Histopathol..

[21] Cai Y., Liu Z., Jia C., Zhao J., Chai S., Li Z., Xu C., Zhang T., Ma Y., Ma C. (2022). Comparison of Sex Differences in Outcomes of Patients with Aneurysmal Subarachnoid Hemorrhage: A Single-Center Retrospective Study. Front. Neurol..

[22] Zamborsky R., Danisovic L. (2020). Surgical Techniques for Knee Cartilage Repair: An Updated Large-Scale Systematic Review and Network Meta-Analysis of Randomized Controlled Trials. Arthrosc. J. Arthrosc. Relat. Surg..

[23] Supartono B. (2018). Hyaline Cartilage Regeneration on Osteochondral Defects by Intraarticular Injection of Human Peripheral Blood CD^34+^ Cells, Hyaluronic Acid and Growth Factor in a Rat Model. Biomed. J. Sci. Tech. Res..

[24] Lubis A.M.T., Wijaya M.T., Priosoeryanto B.P., Saleh R.F., Farqani S. (2022). Comparison of Weekly and Single Dose Intraarticular Recombinant Human Growth Hormone Injection on Cartilage Degeneration in Osteoarthritic Model of White New Zealand Rabbits. J. Exp. Orthop..

[25] Wong K.L., Zhang S., Wang M., Ren X., Afizah H., Lai R.C., Lim S.K., Lee E.H., Hui J.H.P., Toh W.S. (2020). Intra-Articular Injections of Mesenchymal Stem Cell Exosomes and Hyaluronic Acid Improve Structural and Mechanical Properties of Repaired Cartilage in a Rabbit Model. Arthrosc. J. Arthrosc. Relat. Surg..

[26] He L., He T., Xing J., Zhou Q., Fan L., Liu C., Chen Y., Wu D., Tian Z., Liu B. (2020). Bone Marrow Mesenchymal Stem Cell-Derived Exosomes Protect Cartilage Damage and Relieve Knee Osteoarthritis Pain in a Rat Model of Osteoarthritis. Stem Cell Res. Ther..

[27] Sun Q., Zhang L., Xu T., Ying J., Xia B., Jing H., Tong P. (2018). Combined Use of Adipose Derived Stem Cells and TGF-Β3 Microspheres Promotes Articular Cartilage Regeneration In Vivo. Biotech. Histochem..

[28] Kuyinu E.L., Narayanan G., Nair L.S., Laurencin C.T. (2016). Animal Models of Osteoarthritis: Classification, Update, and Measurement of Outcomes. J. Orthop. Surg. Res..

[29] Song X., Liu Y., Chen S., Zhang L., Zhang H., Shen X., Du H., Sun R. (2024). Knee Osteoarthritis: A Review of Animal Models and Intervention of Traditional Chinese Medicine. Anim. Model. Exp. Med..

[30] Deng H., Wang J., An R. (2023). Hyaluronic Acid-Based Hydrogels: As an Exosome Delivery System in Bone Regeneration. Front. Pharmacol..

[31] Riau A.K., Ong H.S., Yam G.H.F., Mehta J.S. (2019). Sustained Delivery System for Stem Cell-Derived Exosomes. Front. Pharmacol..

[32] Kapoor D., Lad C., Vyas R., Patel M. (2016). An overview of viscosupplements: Therapeutic modality for the ailment of osteoarthritis. J. Drug Deliv. Ther..

[33] Zhao F., He W., Yan Y., Zhang H., Zhang G., Tian D., Gao H. (2014). The Application of Polysaccharide Biocomposites to Repair Cartilage Defects. Int. J. Polym. Sci..

[34] Lin X., Zhi F., Lan Q., Deng W., Hou X., Wan Q. (2022). Comparing the Efficacy of Different Intra-Articular Injections for Knee Osteoarthritis: A Network Analysis. Medicine.

[35] Li Q., Yu H., Zhao F., Cao C., Wu T., Fan Y., Ao Y., Hu X. (2023). 3D Printing of Microenvironment-Specific Bioinspired and Exosome-Reinforced Hydrogel Scaffolds for Efficient Cartilage and Subchondral Bone Regeneration. Adv. Sci..

[36] Kim Y.G., Choi J., Kim K. (2020). Mesenchymal Stem Cell-Derived Exosomes for Effective Cartilage Tissue Repair and Treatment of Osteoarthritis. Biotechnol. J..

[37] Xu H., Xu B. (2021). BMSC-Derived Exosomes Ameliorate Osteoarthritis by Inhibiting Pyroptosis of Cartilage via Delivering MiR-326 Targeting HDAC3 and STAT1//NF-ΚB P65 to Chondrocytes. Mediat. Inflamm..

[38] Hayami T., Pickarski M., Wesolowski G.A., Mclane J., Bone A., Destefano J., Rodan G.A., Duong L.T. (2004). The Role of Subchondral Bone Remodeling in Osteoarthritis: Reduction of Cartilage Degeneration and Prevention of Osteophyte Formation by Alendronate in the Rat Anterior Cruciate Ligament Transection Model. Arthritis Rheum..

[39] Lin J., Chen L., Yang J., Li X., Wang J., Zhu Y., Xu X., Cui W. (2022). Injectable Double Positively Charged Hydrogel Microspheres for Targeting-Penetration-Phagocytosis. Small.

[40] Peters A.E., Akhtar R., Comerford E.J., Bates K.T. (2018). The Effect of Ageing and Osteoarthritis on the Mechanical Properties of Cartilage and Bone in the Human Knee Joint. Sci. Rep..

[41] Hu W., Chen Y., Dou C., Dong S. (2021). Microenvironment in Subchondral Bone: Predominant Regulator for the Treatment of Osteoarthritis. Ann. Rheum. Dis..

[42] Xiao Z., He J., Su G., Chen M., Hou Y., Chen S., Lin D. (2018). Osteoporosis of the Vertebra and Osteochondral Remodeling of the Endplate Causes Intervertebral Disc Degeneration in Ovariectomized Mice. Arthritis Res. Ther..

[43] Cosenza S., Ruiz M., Toupet K., Jorgensen C., Noël D. (2017). Mesenchymal Stem Cells Derived Exosomes and Microparticles Protect Cartilage and Bone from Degradation in Osteoarthritis. Sci. Rep..

[44] Jin Y., Xu M., Zhu H., Dong C., Ji J., Liu Y., Deng A., Gu Z. (2021). Therapeutic Effects of Bone Marrow Mesenchymal Stem Cells--derived Exosomes on Osteoarthritis. J. Cell Mol. Med..

[45] Qi H., Liu D.-P., Xiao D.-W., Tian D.-C., Su Y.-W., Jin S.-F. (2019). Exosomes Derived from Mesenchymal Stem Cells Inhibit Mitochondrial Dysfunction-Induced Apoptosis of Chondrocytes via P38, ERK, and Akt Pathways. Vitr. Cell. Dev. Biol. Anim..

