# Supplementary material for: Intra-Articular Injection of Adipose-Derived-MSC Exosomes and Hyaluronic Acid in Sheep Knee Osteoarthritic Models Enhances Hyaline Cartilage Regeneration
Source: Biomedicines. 2025 Dec 12;13(12):3070. doi: 10.3390/biomedicines13123070 (PMC12730780; doi:10.3390/biomedicines13123070)
Supplement: Supplementary file 1 [file biomedicines-13-03070-s001.zip › biomedicines-3950935-Supplementary Materials.pdf]

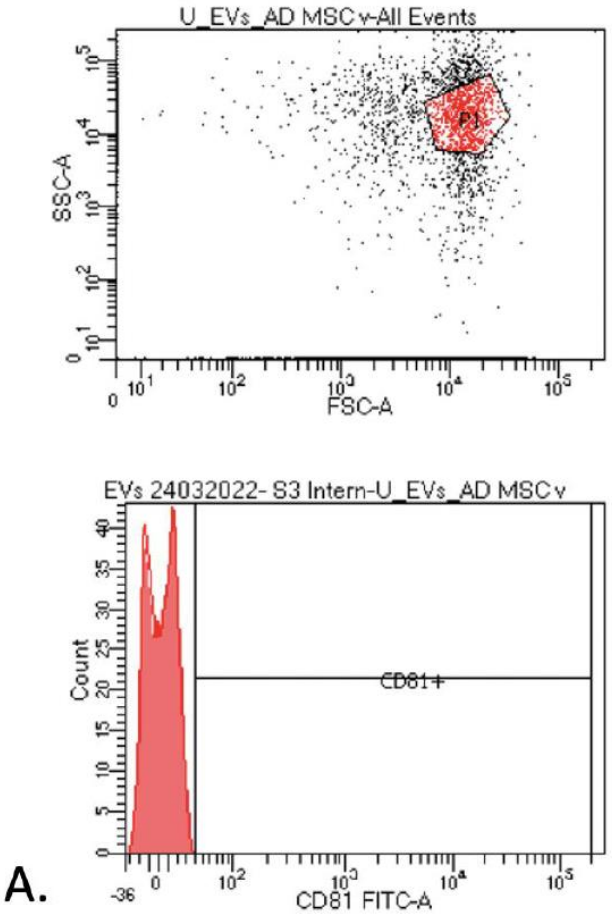

B.

Tube: U_EVs_AD MSC v

Population	#Events	%Parent	%Total
All Events	5,147	####	100.0
P1	845	16.4	16.4
CD81+	0	0.0	0.0

Experiment Name:	RDA-PINK-compensated Annexin V_26-08-19
Specimen Name:	EVs 24032022- S3 Intern
Tube Name:	U_EVs_AD MSC v
Record Date:	Mar 24, 2022 2:55:07 PM
$OP:	Administrator
GUID:	272cf3e8-0427-4c72-97e0-4315cafd6235

Population	#Events	%Parent	CD81 FITC-A Mean	CD81 FITC-A Median
All Events	5,147	####	-1	-1
P1	845	16.4	-1	-1
CD81+	0	0.0	####	####

Figure S1. Characterization of Adipose SPM Exosomes Using Flow Cytometry CD63 and CD81 in unstained sample. (A.) Shows the gating strategy for the exosome population within the pentagonal curve under unstained conditions. Unstained samples were not incubated with CD81, so in image (B.) below, it can be seen that the subpopulation of exosomes not treated with antibodies is 0%.

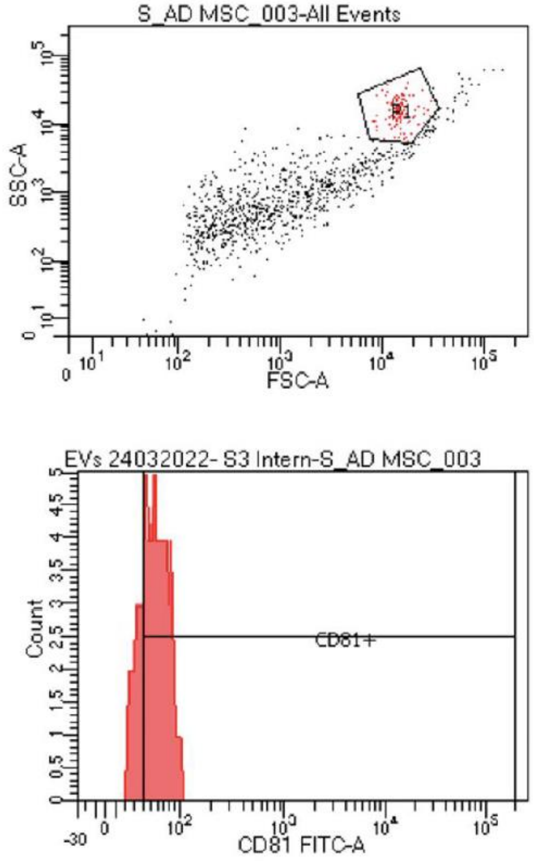

A.

Tube: S_AD MSC_003

Population	#Events	%Parent	%Total
All Events	1,021	####	100.0
P1	125	12.2	12.2
CD81+	84	67.2	8.2

Experiment Name:	RDA-PINK-compensated Annexin V_26-08-19
Specimen Name:	EVs 24032022- S3 Intern
Tube Name:	S_AD MSC_003
Record Date:	Mar 24, 2022 3:47:15 PM
$OP:	Administrator
GUID:	57d4a700-a6d4-4223-9faa-be0914043021

Population	#Events	%Parent	CD81 FITC-A Mean	CD81 FITC-A Median
All Events	1,021	####	27	12
P1	125	12.2	59	50
CD81+	84	67.2	74	62

B.

Figure S2. Characterization of Adipose SPM Exosomes Using enrichment of CD63 antibody and CD81 antibody Flow Cytometry. (A.) Shows the gating strategy for the exosome population within the pentagonal curve. (B.) Shows the histogram of the double-positive CD63+ and CD81+ exosome population. (B.) Shows the percentage of the CD63+ and CD81+ exosome subpopulation at 67.2%.

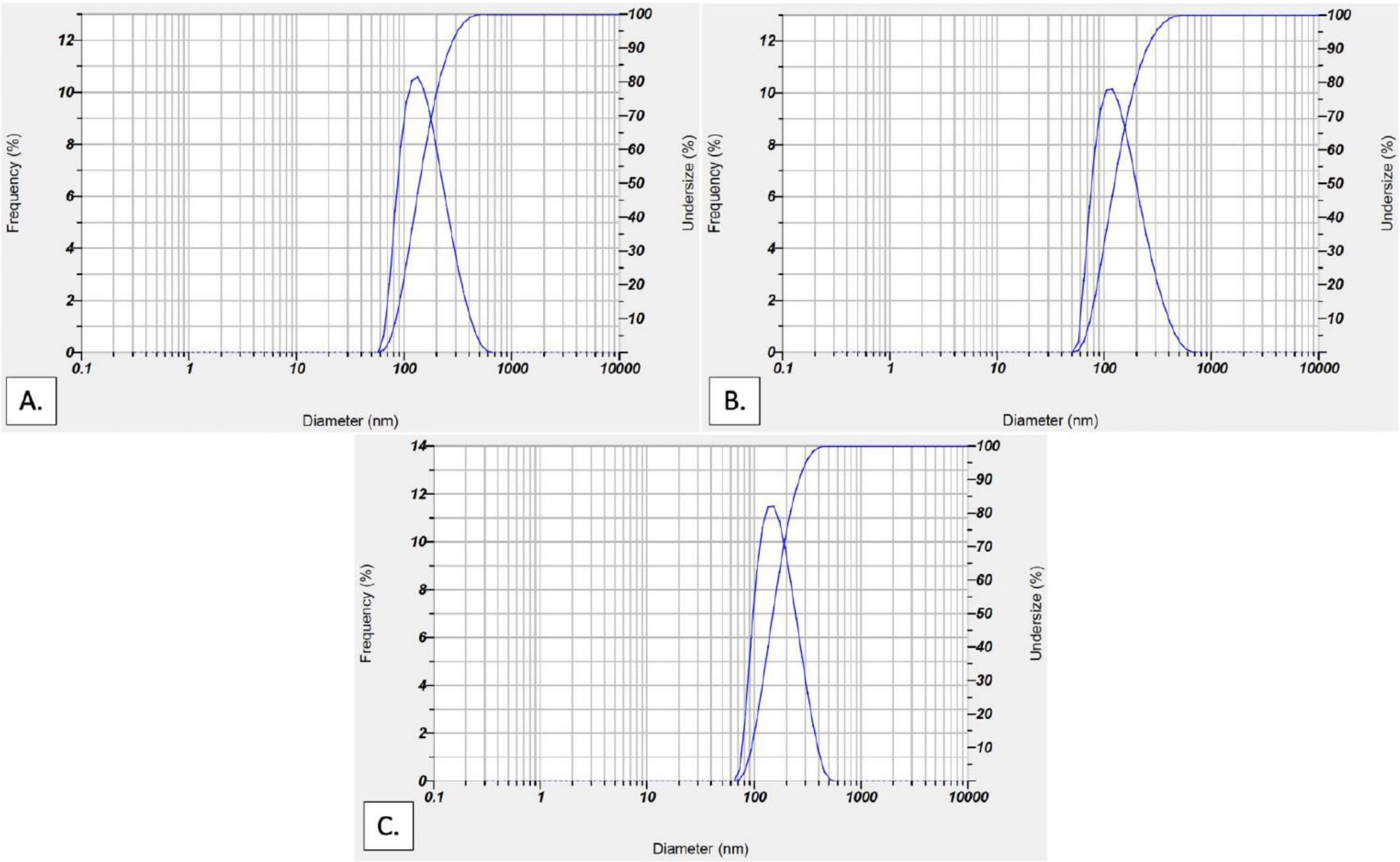

Figure S3. Characterization of Adipose SPM Exosomes Based on Particle Size with PSA. A single parabolic graph shows the normal distribution of particle sizes with a peak representing the highest frequency of particle sizes, while the legs of the parabolic curve show the minimum and maximum values of particle sizes. The X-axis represents particle size and the Y-axis represents the frequency of particle distribution. The plateau graph shows the total number of particles that have been analyzed. There are 3x particle size repetitions shown in the figure, (A) Series 1, (B) Series 2, and (C) Series 3.
